# The RNA-Binding Protein NELFE Promotes Gastric Cancer Growth and Metastasis Through E2F2

**DOI:** 10.3389/fonc.2021.677111

**Published:** 2021-07-06

**Authors:** Changyu Chen, Qiang Zheng, Shubo Pan, Wenzheng Chen, Jianfeng Huang, Yi Cao, Yi Tu, Zhengrong Li, Changjun Yu, Zhigang Jie

**Affiliations:** ^1^ Department of Gastrointestinal Surgery, The First Affiliated Hospital of Nanchang University, Nanchang, China; ^2^ Department of Anesthesiology (High-Tech Branch), The First Affiliated Hospital of Anhui Medical University, Hefei, China; ^3^ Department of General Surgery, The First Affiliated Hospital of Anhui Medical University, Hefei, China; ^4^ Department of General Surgery, The Second Affiliated Hospital of Anhui Medical University, Hefei, China; ^5^ Department of Pathology, The First Affiliated Hospital of Nanchang University, Nanchang, China

**Keywords:** NELFE, E2F2, gastric cancer, proliferation, metastasis

## Abstract

Worldwide, the incidence rate of gastric cancer ranks fifth, and the mortality rate of gastric cancer ranks third among all malignant tumors. However, the pathogenesis of gastric cancer remains poorly understood. In this study, we demonstrated that the expression level of NELFE is higher in human gastric cancer tissues than in adjacent nontumor tissues. A high level of NELFE is associated with worse postoperative overall survival (OS) and relapse-free survival (RFS) rates in patients with gastric cancer. Moreover, the expression of NELFE is correlated with high tumor grade and lymph node metastasis in gastric cancer patients. Knockdown of NELFE dramatically inhibits the cell proliferation and metastasis of gastric cancer xenografts *in vivo*. Furthermore, we found that NELFE binding to the 3’UTR of E2F2 affects the mRNA stability of E2F2 to regulate the expression level of E2F2. In gastric cancer, E2F2 also acts as an oncogene to inhibit the proliferation and migration of gastric cancer cells by knocking down the expression level of E2F2. However, overexpressing E2F2 in cells with NELFE knockdown significantly reverses the inhibition of cell proliferation and migration induced by NELFE knockdown. Therefore, NELFE at least partially functions as an oncogene through E2F2. Moreover, CIBERSORTx analysis of the proportion of tumor-infiltrating immune cells (TICs) revealed that immune cells are correlated with NELFE and E2F2 expression, suggesting that NELFE and E2F2 might be responsible for the preservation of the immunodominant status for gastric cancer. In conclusion, NELFE acts as an oncogene in gastric cancer and can be used as a potential therapeutic target.

## Introduction

Gastric cancer is one of the leading causes of cancer-related mortality worldwide (1, 2). Patients with gastric cancer are usually diagnosed in advanced stages. At present, the treatment methods for gastric cancer are also relatively limited, mainly surgical resection and adjuvant chemotherapy or radiotherapy (3). However, the recurrence rate of gastric cancer is still high, and the prognosis is poor (2). Although the prevalence and etiopathogenesis of gastric cancer exist with geographic differences (4), the 5-year survival rate of gastric cancer is also low compared to that of other malignant tumors worldwide. Therefore, it is necessary to further study the molecular mechanisms that promote the progression of gastric cancer to develop new diagnostic or prognostic indicators, as well as treatment strategies to improve clinical outcomes.

RNA-binding proteins play a multifaceted and crucial role in posttranscriptional gene regulation processes, such as RNA splicing, transport, translation, localization and stability (5–7). In recent years, an increasing number of studies have shown that RNA-binding proteins play an important role in the progression and drug resistance of tumors (8–13). Negative elongation factor complex member E (NELFE) is an RNA-binding protein. Dang et al. (14) reported that NELFE is an oncogenic protein that may cause transcriptome imbalance in hepatocellular carcinoma (HCC) by regulating MYC signaling and the NELFE-dependent MYC target (NDMT) gene signature to predict a unique subtype of HCC. Borisova et al. (15) reported that MK2 phosphorylates NELFE on serine 115 and that phosphorylation of NELFE can promote the rapid dissociation of 14-3-3 and the NELF complex from chromatin, accompanied by RNA polymerase II extension. Yu et al. (16) demonstrated that NELFE promoted gastric cancer progression by regulating CSNK2B. To the best of our knowledge, the intrinsic mechanism of NELFE with respect to promoting human gastric cancer is still unclear and further investigations are necessary.

A growing body of studies has demonstrated the importance of the tumor microenvironment (TME) in tumor development (17). As an important structural component of the TME, immune cells play important roles in tumor growth and progression. Increasing evidence has shown that tumor-infiltrating immune cells (TICs) in the TME directly or indirectly promote gastric tumorigenesis and the response to chemotherapy (18). However, the function of NELFE in gastric cancer and its role in TICs are still unclear and need to be studied.

In this study, we determined the tumor-promoting effect of NELFE in human gastric cancer. Overexpression of NELFE was observed in human gastric cancer tissues compared with adjacent nontumor tissues. High levels of NELFE were associated with low overall survival (OS) and low recurrence-free survival (RFS) in human gastric cancer patients. The results showed that knocking down the expression of NELFE can inhibit the proliferation and migration of gastric cancer cells *in vitro* and *in vivo*. Transcriptome sequencing data showed that knocking down the expression of NELFE caused significant changes in the expression of a number of downstream genes, of which we focused on the E2F2 gene. It was determined that the NELFE protein could directly bind to the 3’UTR of E2F2 and promote the expression of E2F2. Restoring the expression of E2F2 obviously reversed the cell proliferation and migration inhibition caused by NELFE knockdown. CIBERSORT analysis of the proportion of TICs revealed that immune cells were correlated with NELFE and E2F2 expression. Therefore, NELFE acts as an oncogene in human gastric cancer cells, and targeting NELFE can be considered a potential method for gastric cancer treatment.

## Materials and Methods

### Tissue Samples and Patients

Human gastric cancer tissues and adjacent nontumor tissues were collected at the Department of Pathology at the First Affiliated Hospital of Anhui Medical University (Hefei, Anhui, China) between 2013 and 2014. Patients with other diseases or special treatments were excluded from treatment before surgery. These gastric cancer patients were followed up for more than 5 years before the end of follow-up on December 31, 2019. 32 pairs of fresh gastric cancer tissues and adjacent nontumor tissues were collected at the Department of Gastrointestinal Surgery at the First Affiliated Hospital of Anhui Medical University in May 2019 and stored in liquid nitrogen. Clinical pathological TNM staging was carried out using standard methods (19). This study plan was approved by the institutional review boards of Anhui Medical University and was carried out in accordance with the code of ethics of the World Medical Association (Declaration of Helsinki). All patients had signed an informed consent form.

### Immunohistochemistry

Tissue samples were paraffin embedded, sliced, and used for immunohistochemical testing according to standard procedures. Sections stained for immunohistochemistry were evaluated by pathologists using an Olympus microscope (Olympus, Japan). Antibodies against NELFE (1:100, 10705-1-AP, Proteintech Group, USA) and E2F2 (1:100, sc-9967, Santa Cruz, USA) were used.

### Western Blot Analysis

The protein levels of NELFE and E2F2 in both cell lines and fresh human tissues were detected using western blotting. In short, 35 μg of protein was separated by a 10% SDS-PAGE gel and then transferred to a PVDF membrane (Millipore, USA). The membrane was blocked with 5% BSA for 1 hour at room temperature. Next, the membrane was incubated with NELFE antibody (1:1,000, 10705-1-AP, Proteintech Group, USA), E2F2 antibody (1:1,000, sc-9967, Santa Cruz, USA), or β-actin antibody (1:5,000, 66009-1-Ig, Proteintech Group, USA) overnight at 4°C. The membrane was then incubated with the secondary antibody (1:5,000, SA00001-2, Proteintech Group, USA) for 1 hour at room temperature. The protein bands were visualized with a chemiluminescence system (EMD Millipore). β-Actin was used as a control.

### Cell Lines and Culture

Human gastric cancer cells (BGC-823, AGS, SGC-7901, HGC-27, MKN-45, and MGC-803) and liver cancer cells (Huh-1 and Hep3B) were obtained from Shanghai Cell Bank (Shanghai, China) or ATCC (the American Type Culture Collection). All of these cell lines were cultured at 5% CO2 and 37°C in a humidified atmosphere as recommended.

### Cell Functional Assays

In this study, an MTT assay was carried out to evaluate cell viability. First, 1000 cells were plated into 96-well plates. From day 1 to day 5, 20 µL of MTT solution (5 mg/mL) was added to each well, and 570 nm wavelength was measured by a 96-well plate reader as described in a previous study.

For the cell colony formation assay, 1000 cells per well were seeded into 6-well plates, and colony formation was examined 10 days later as described previously.

For the migration assay, 5-10 × 10^4^ BGC-823 and AGS cells were added to the top 8-µm chamber without Matrigel. For invasion assays, 10-20 × 10^4^ cells were added to a Matrigel-coated upper chamber. The lower chambers were filled with medium containing 10-20% serum. After 24-48 hours of incubation, the inserts were rinsed with PBS and stained with 0.1% crystal violet solution for 5 min. Images were taken by an Olympus IX-70 microscope.

### Xenograft Assays

Five-week-old male nude mice were used in xenograft assays. A total of 5 × 10^6^ BGC-823 cells were mixed with the same volumes of Matrigel and injected subcutaneously into the flanks or tail veins of the animals. The volumes of tumors were continuously measured every three days after one week.

### RT-qPCR

The mRNA levels of NELFE and E2F2 were examined using RT-quantitative PCR (RT-qPCR). GAPDH was used as control. The primers used in this study were as follows: NELFE (F: CAGATGGAGAAGAGGCAGAGG, R: GTTCAGGGAATGAATCCGACC), E2F2 (F: GAGCAGGCCTTGGACCAG, R: CCCTTGGGTGCTCTTGAG) and GAPDH (F: CTGCCTCTACTGGCGCTG, R: GGTCAGGTCCACCACTGAC).

### mRNA Decay Assay

Gastric cancer cells were treated with 10 μg/mL actinomycin D. RNA was collected at 0, 2, 4, 6, and 8 hours, and the mRNA level of E2F2 was quantified by real-time fluorescent qPCR. GAPDH served as an endogenous control.

### mRNP Immunoprecipitation for NELFE

The anti-NELFE antibody was used to capture the NELFE protein-E2F2 mRNA complex, and RT-qPCR was used to quantify the level of E2F2 mRNA. IgG was used as a control. The mRNA level of the negative control gene GAPDH was also determined.

### Luciferase Assays

The 3′-UTRs of the human E2F2 were cloned into the dual-luciferase expression vector, psiCHECK2 vector. BGS-823 and AGS cells were plated in 24-well plates. Cells of 50% confluence were transfected using Lipofectamine 3000 (Invitrogen). Cell extracts were prepared after 48hs posttransfection with 100 ng plasmid and the luciferase activity was examined by the Dual Luciferase Assay System (Promega).

### Biotin Pulldown Analysis

The biotinylated E2F2 entire 5’UTR, CDS and 3’UTR RNAs were obtained by chemical synthesis followed by biotin labeling. We purchased the above biotinylated RNA from a biological company (Sangon biotech, Shanghai, China). 3 μg biotinylated RNAs were incubated with whole-cell lysates (200 μg/sample) for 1 h at room temperature, and then complexes were isolated with streptavidin-coupled Dynabeads (#65305, Invitrogen). The association of NELFE with these RNAs was detected by Western blot analysis.

### Raw Data of TICs

Transcriptome RNA sequencing data of 407 STAD cases (normal samples, 32 cases; tumor samples, 375 cases) and the corresponding clinical data were downloaded from TCGA database (https://portal.gdc.cancer.gov/) with level 3.

### Heatmaps

Heatmaps of differentially expressed genes (DEGs) were generated by R language with the package pheatmap.

### TIC Profile

The CIBERSORTx (20) computational method was applied to estimate the TIC abundance profile in all tumor samples.

### Statistical Analyses

Data from cell proliferation and metastasis assays were analyzed using unpaired two-tailed t-tests. We used GraphPad Prism 8.02 software to reassess the correlation between NELFE and E2F2. Correlation analysis of clinicopathological parameters was performed using the Pearson chi-square test and Spearman rank correlation test. P<0.05 was considered statistically significant.

## Results

### High Expression of NELFE in Gastric Cancer Patients Indicates a Worse Survival Outcome for Patients

**Figure 1:**
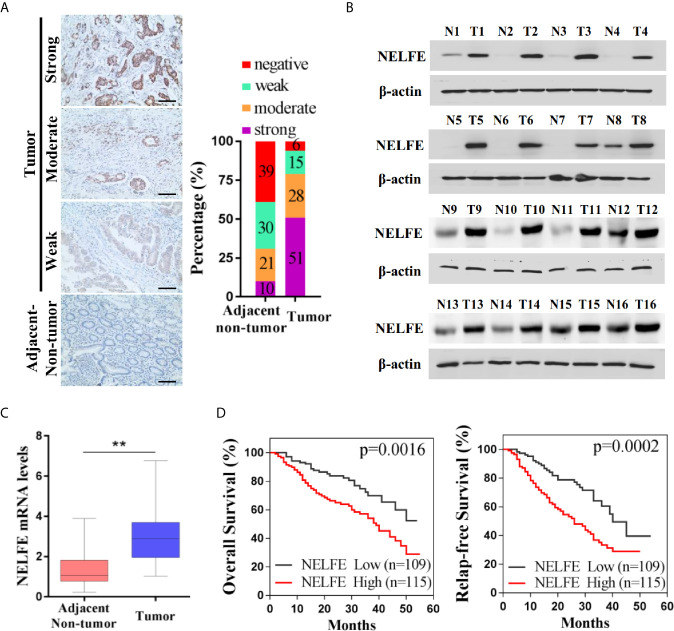
NELFE was overexpressed in gastric cancer and associated with poor survival rates in gastric cancer patients. **(A)** The expression levels of NELFE in 236 pairs of gastric cancer tissues and normal gastric tissues were examined by immunohistochemical staining. **(B, C)** The expression levels of NELFE in 32 pairs of fresh tumor tissues and adjacent nontumor tissues were detected by western blotting and qRT-PCR. **(D)** The overall survival (OS) rates and relapse-free survival (RFS) rates in gastric cancer patients with high NELFE expression and low NELFE expression were analyzed by Kaplan-Meier curves. **P < 0.01.

We collected tumor and adjacent nontumor tissue samples from 224 patients with gastric cancer. As shown by immunohistochemical examination in **Figure 1A**, 10% of patients with gastric cancer had strong NELFE protein expression in adjacent tissues, 21% had moderate NELFE expression, 30% had weak NELFE positive expression, and 39% were NELFE negative. The corresponding percentages in tumor tissues were 51%, 28%, 15% and 6%, respectively. The expression levels of NELFE in 32 pairs of fresh tumor tissues and adjacent nontumor tissues were detected by western blot and qRT-PCR assays. Correspondingly, the protein and RNA levels of NELFE in gastric cancer tissues were significantly higher than those in adjacent nontumor tissues (**Figures 1B, C** and **Supplementary Figure S1**). The correlation between NELFE expression and survival was analyzed in 224 patients with gastric cancer. As shown in **Figure 1D**, compared with gastric cancer patients with high NELFE levels, gastric cancer patients with low NELFE levels had significantly higher OS rates (P = 0.0016) and RFS rates (P = 0.0002). To understand the clinical implications of the increased expression, we examined the correlation of NELFE with the clinicopathologic features of GC. The higher NELFE expression was observed to be associated with tumor size (p < 0.01), differentiation of tumor (p < 0.01), TNM stage (p < 0.001) and lymph node metastasis (p < 0.001) of GC (**Supplementary Table S1**). The above results show that NELFE may be a potential oncogene in gastric cancer.

### NELFE Promotes the Proliferation of Human Gastric Cancer Cells

**Figure 2:**
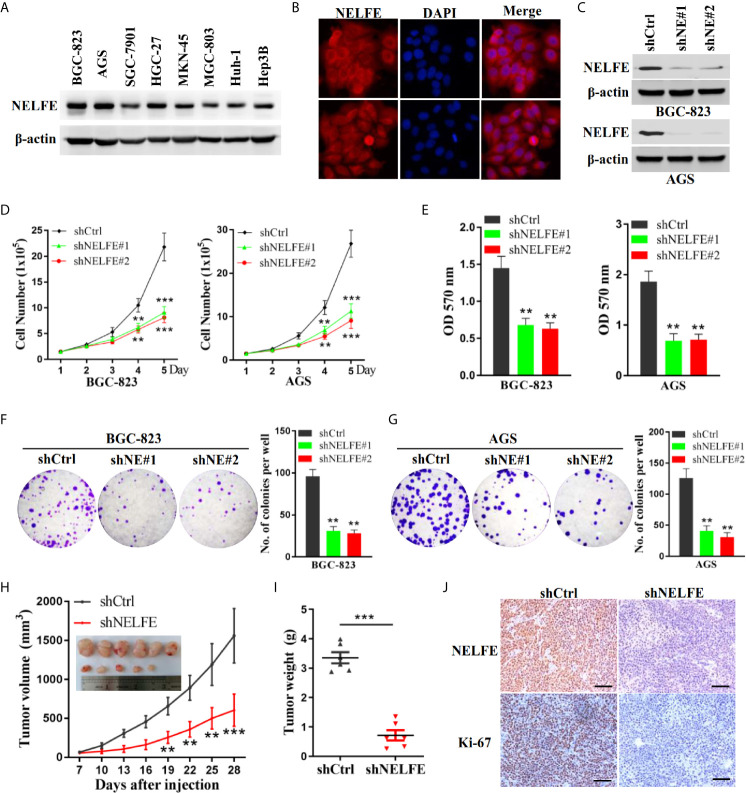
NELFE promoted the proliferation of human gastric cancer cells. **(A)** The expression levels of NELFE were examined by western blotting in liver cancer cells (Huh-1 and Hep3B) and gastric cancer cells (BGC-823, AGS, SGC-7901, HGC-27, MKN-45, and MGC-803). **(B)** The distribution of the NELFE protein in the cells was examined by immunofluorescence experiments. BGC-823 and AGS gastric cancer cells were transfected with sh-NELFE#1, sh-NELFE#2, or sh-control. **(C)** The protein expression level of NELFE was detected by western blotting. Actin was used as control. **(D, E)** Cell viability was measured by the MTT assay. **(F, G)** A cell colony formation assay was performed in BGC-823 and AGS cells. **(H, I)** Five-week-old male nude mice were used in xenograft assays, and BGC-823 cells were mixed with Matrigel and injected subcutaneously into the flanks. **(J)** The proportion of ki67-expressing cells in xenograft tumors was examined by immunohistochemical examination. **P < 0.01, ***P < 0.001.

Previous studies have shown that NELFE is expressed at high levels in Huh-1 and Hep3B liver cancer cells. We tested the expression level of NELFE in gastric cancer and liver cancer cell lines. As shown in **Figure 2A**, the NELFE protein was generally expressed in gastric cancer cells, and its protein expression level was high in some gastric cancer cell lines, especially in BGC-823 and AGS cells. On the other hand, NELFE was relatively low expressed in SGC-7901 and MGC-803. Therefore, these four cell lines were selected for further functional studies. Next, we investigated the distribution of the NELFE protein in these cells by immunofluorescence experiments and found that the NELFE protein was clearly observed in the cytoplasm (**Figure 2B**). As shown in **Figure 2C** and **Supplementary Figure S2A**, the protein expression level of NELFE was significantly decreased or increased after shRNA-mediated knockdown or NELFE-overexpression plasmid-mediated overexpression of NELFE. The results of cell proliferation assays showed that the number of cells was significantly lower in the NELFE-knockdown group than the control group (**Figure 2D**). Next, cell viability was measured using the MTT assay. The results showed that cell viability was significantly inhibited after NELFE knockdown *in vitro* (**Figure 2E**), and the number of colonies formed by AGS and BGC-823 gastric cancer cells decreased significantly (**Figures 2F, G**). Conversely, overexpression of NELFE facilitated cell proliferation (**Supplementary Figure S2B**), cell viability(**Supplementary Figure S2C**) and the number of colonies formed (**Supplementary Figure S2D**). To further study the function of NELFE *in vivo*, we injected BGC-823 cells with stable knockdown of NELFE and control cells into the abdominal flanks of nude mice. The results showed that compared with those in the control group, the tumors formed by BGC-823 cells with NELFE knockdown were smaller and lighter (**Figures 2H, I**), and the proportion of ki67-positive cells in these tumors was significantly lower (**Figure 2J**). In short, knocking down NELFE significantly inhibited the growth of gastric cancer cells *in vitro* and their tumor formation ability *in vivo*.

### NELFE Promotes the Metastasis of Human Gastric Cancer Cells

**Figure 3:**
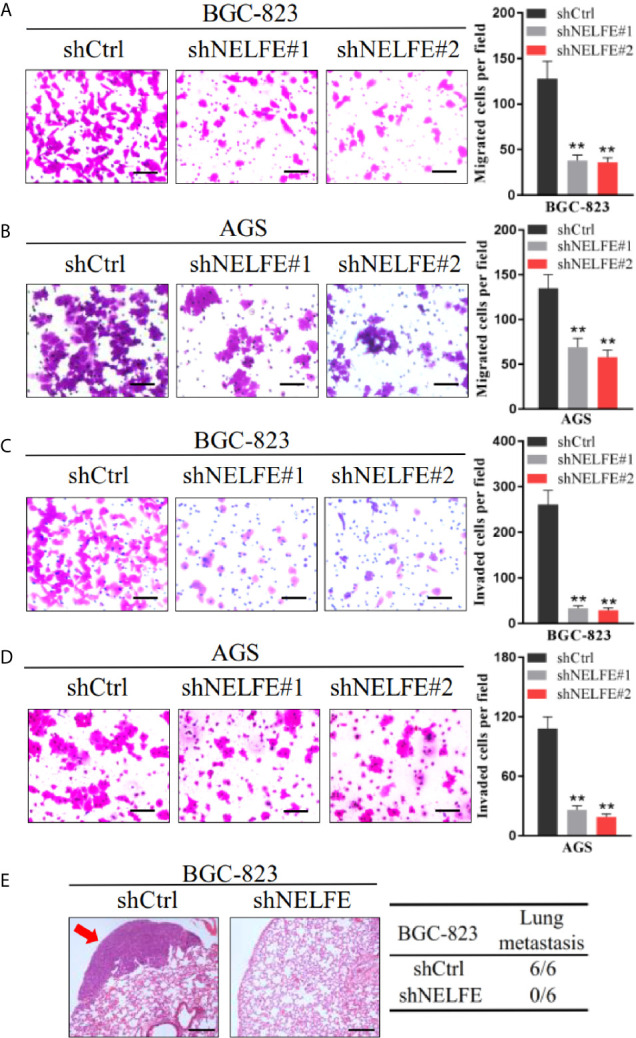
NELFE promoted metastasis of human gastric cancer cells. **(A, B)** Transwell migration and **(C, D)** Invasion assays. **(E)**, Five-week-old male nude mice were used in xenograft assays, and BGC-823 cells were injected into the tail vein. Representative images of H&E staining of lungs and incidence of lung metastasis from mice inoculated with BGC-823 cells. **P < 0.01.

To explore the effect of NELFE on cell migration and invasion, we tested the effect of NELFE knockdown and overexpression on cell migration and invasion through Transwell experiments. As shown in **Figures 3A, B**, knockdown of NELFE in BGC-823 and AGS cells inhibited their migration. Then, we tested the effect of NELFE knockdown on cell invasion and observed that cell invasion was also inhibited (**Figures 3C, D**). Overexpression of NELFE promoted cells migration (**Supplementary Figure S2E**) and invasion (**Supplementary Figure S2F**). Furthermore, we used tail vein injection of BGC-823 cells to study the effect of NELFE on the metastatic ability of gastric cancer cells *in vivo*. The results showed that in the NELFE-knockdown group, no tumor metastases were observed in the lungs of the mice (0/6), while lung metastases were observed in each mouse in the control group (6/6) (**Figure 3E**). Therefore, NELFE is necessary for the metastasis of human gastric cancer cells.

### E2F2 Is Regulated by NELFE in Human Gastric Cancer Cells

**Figure 4:**
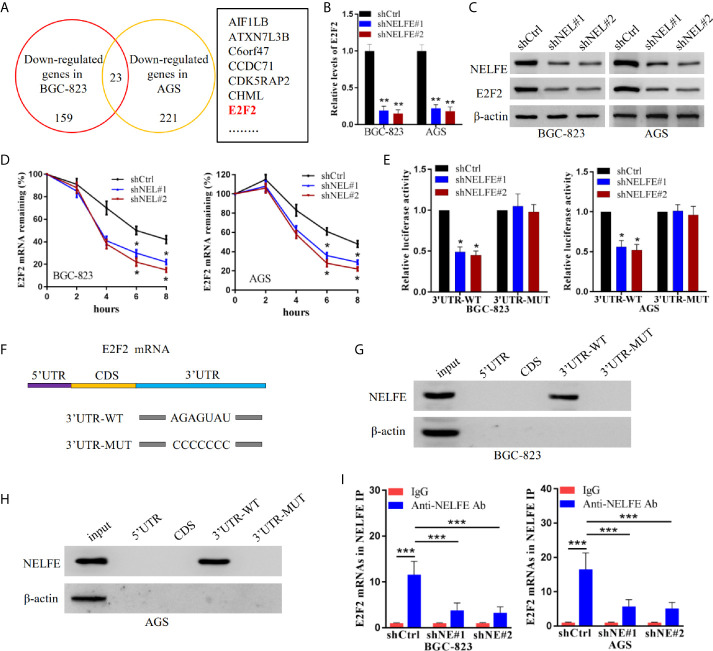
E2F2 was regulated by NELFE in human gastric cancer cells. **(A)** Graph of 23 genes whose expression decreased in BGC-823 and AGS cells after NELFE knockdown, as demonstrated by RNA sequencing. **(B, C)** The expression levels of E2F2 were detected by qRT-PCR and western blotting when NELFE was knocked down. **(D)** The mRNA decay rate of E2F2 in NELFE-knockdown BGC-823 and AGS cells was detected by the mRNA decay assay. **(E)** Change in the E2F2 3’UTR luciferase reporter activity with the consumption of NELFE. **(F)** Schematic depiction of the E2F2 5’UTR, CDS and 3’UTR, as well as the biotinylated RNAs synthesized for use in biotin pulldown analysis. The interaction of NELFE protein with each biotinylated E2F2 5’UTR, CDS, wild-type 3’UTR, and mut-type 3’UTR RNA segment was analyzed by western blotting in BGC-823 **(G)** and AGS **(H)** cells, with β-actin as input control. **(I)** Enrichment of E2F2 mRNA was conducted by the mRNP immunoprecipitation assay. *P < 0.05, **P < 0.01, ***P < 0.001.

To explore the downstream regulatory genes of NELFE, we used RNA sequencing to search for changes in gene expression after knocking down NELFE. Sequencing results showed that there were 23 genes whose expression decreased in BGC-823 and AGS cells after knocking down NELFE (**Figure 4A**). Among them, the E2F2 gene drew our attention. The results of real-time fluorescence qPCR confirmed that the expression of E2F2 decreased significantly when NELFE was knocked down (**Figure 4B**). The western blotting results also confirmed that the protein expression of E2F2 decreased significantly when NELFE was knocked down (**Figure 4C**). As shown in **Figure 4D**, the mRNA decay rate of E2F2 decreased dramatically in both NELFE-knockdown BGC-823 and AGS cells. Depletion of NELFE significantly decreased the luciferase activity of the E2F2 3’UTR reporter (**Figure 4E**). Moreover, the biotin-labeled E2F2 3’UTR could bind to the NELFE protein, while other regions and mutants labeled with biotin could not bind to the NELFE protein (**Figures 4F–H**). In the RNP-IP assay, the anti-NELFE antibody dramatically enriched E2F2 mRNA compared with the control IgG (**Figure 4I**). Thus, E2F2 is regulated by NELFE through posttranscriptional 3’UTR binding.

### E2F2 Promotes the Growth and Metastasis of Human Gastric Cancer Cells

**Figure 5:**
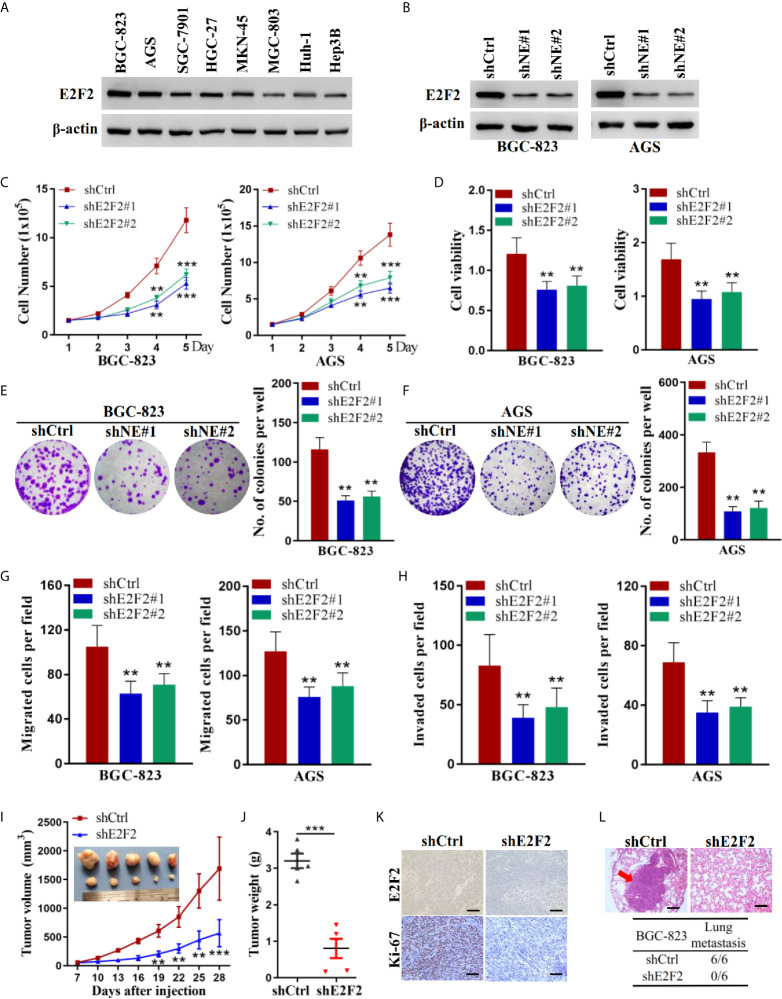
E2F2 promoted the growth and metastasis of human gastric cancer cells. **(A)** Expression levels of E2F2 were examined by western blot in liver cancer cells (Huh-1 and Hep3B) and gastric cancer cells (BGC-823, AGS, SGC-7901, HGC-27, MKN-45, and MGC-803). BGC-823 and AGS gastric cancer cells were transfected with sh-E2F2#1, sh-E2F2#2, or sh-control. **(B)** The protein expression level of E2F2 was detected by western blotting. Actin was used as control. **(C, D)** Cell viability was measured by the MTT assay. **(F, G)** A cell colony formation assay was performed in BGC-823 and AGS cells. **(E, F)** A cell colony formation assay was performed in BGC-823 and AGS cells. **(G)** Transwell migration and **(H)** invasion assays. **(I, J)** Five-week-old male nude mice were used in xenograft assays, and BGC-823 cells were mixed with Matrigel and injected subcutaneously into the flanks. **(K)** The proportion of ki67-expressing cells in xenograft tumors was examined by immunohistochemical staining. **(L)** BGC-823 cells were injected into the tail vein. Representative images of H&E staining of lungs and incidence of lung metastasis from mice inoculated with BGC-823 cells. **P < 0.01, ***P < 0.001.

We examined the protein levels of E2F2 in human gastric cancer cells. Similar to NELFE, the E2F2 protein was ubiquitously expressed in gastric cancer cells, and the expression level was higher in BGC-823 and AGS cells (**Figure 5A**). We knocked down the expression of E2F2 by shRNA (**Figure 5B**). Knockdown of E2F2 dramatically decreased the total number (**Figure 5C**), viability (**Figure 5D**), colony formation (**Figures 5E, F**), migration (**Figure 5G**), and invasion (**Figure 5H**) of both BGC-823 and AGS cells. To further study the function of E2F2 *in vivo*, we injected BGC-823 cells with stable E2F2 knockdown or control cells into the abdominal flanks of nude mice. The results showed that compared with those formed by control cells, the tumors formed by BGC-823 cells with E2F2 knockdown were smaller and lighter (**Figures 5I, J**). Furthermore, we used tail vein injection of BGC-823 cells to study the effect of E2F2 on the metastatic ability of gastric cancer cells *in vivo*. The results showed that in the E2F2-knockdown group, no tumor metastases were observed in the lungs of the mice (0/6), while metastases were observed in the lungs of each mouse in the control group (6/6) (**Figures 5K, L**). Therefore, E2F2 also promotes the growth and metastasis of human gastric cancer cells *in vitro* and *in vivo*.

### E2F2 Overexpression Reversed the Cancer-Promoting Function of NELFE

**Figure 6:**
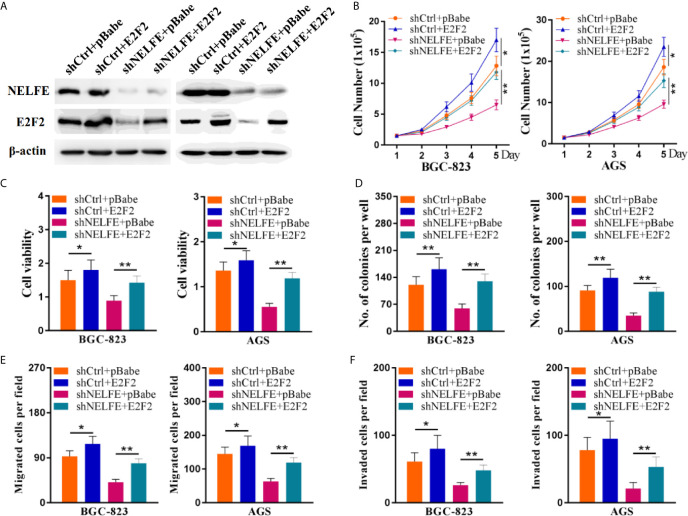
E2F2 overexpression reversed the cancer-promoting effect of NELFE. Overexpression of E2F2 plus knockdown of NELFE rescues the phenotype induced by NELFE knockdown. **(A)** The restoration of E2F2 expression in BGC-823 and AGS cells was confirmed by western blotting. **(B)**, Proliferation curves of BGC-823 and AGS cells transfected with the control shRNA plus pBabe, shRNA plus E2F2, sh-NELFE plus pBabe and sh-NELFE plus E2F2. **(C, D)** MTT and colony formation assays of BGC-823 and AGS cells transfected with the control shRNA plus pBabe, shRNA plus E2F2, sh-NELFE plus pBabe and sh-NELFE plus E2F2. **(E)** Transwell migration and **(F)** invasion assays of BGC-823 and AGS cells transfected with the control shRNA plus pBabe, shRNA plus E2F2, sh-NELFE plus pBabe and sh-NELFE plus E2F2. *P < 0.05, **P < 0.01.

To verify whether E2F2 mediates the cancer-promoting effect of NELFE, we restored the expression level of E2F2 through an expression plasmid lacking the E2F2 3’UTR. The restoration of E2F2 expression in BGC-823 and AGS cells was confirmed by western blotting (**Figure 6A**). Consistent with previous results, knockdown of NELFE dramatically decreased the total number, viability, colony formation, migration, and invasion of BGC-823 and AGS cells (**Figures 6B–F**). In addition, these decreases were abated by the forced expression of E2F2 (**Figures 6B–F**). Therefore, E2F2 is necessary for NELFE to promote human gastric cancer.

### E2F2 Expression Is Elevated In Gastric Cancer and Is Positively Correlated With NELFE Expression

**Figure 7:**
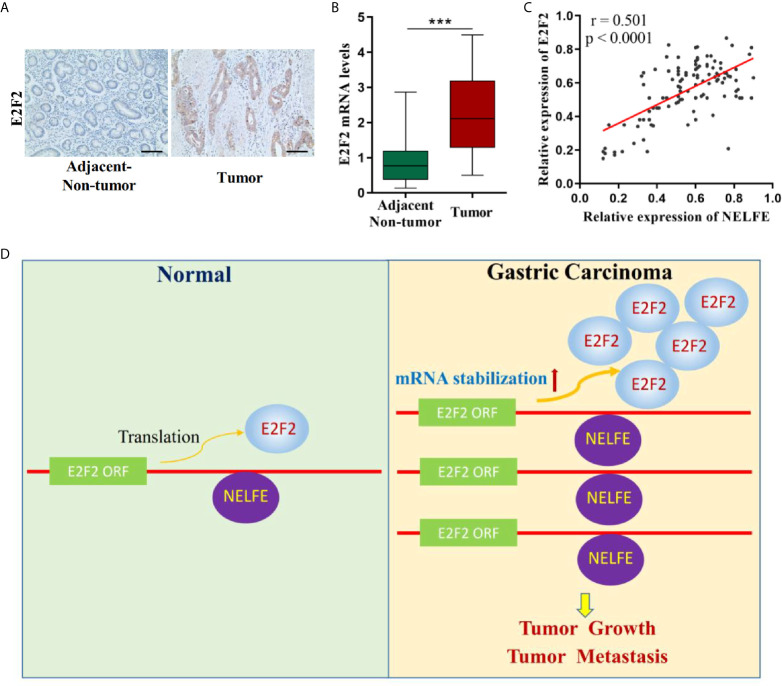
E2F2 expression was elevated in gastric cancer and is positively correlated with NELFE. **(A)** Expression levels of E2F2 in gastric cancer tissues and normal gastric tissues were examined by immunohistochemical examination. **(B)** The expression levels of E2F2 in 32 pairs of fresh tumor tissues and adjacent nontumor tissues were detected by qRT-PCR. **(C)** The correlation between NELFE and E2F2. **(D)** The molecular mechanism of upregulation of E2F2 mRNA through NELFE in promoting gastric cancer pathogenesis. ***P < 0.001.

The levels of E2F2 in gastric tissues were examined. As shown in **Figures 7A, B**, compared with that in adjacent nontumor tissues, the expression level of E2F2 in tumor tissues was higher. In addition, after analyzing the correlation between NELFE and E2F2, we found that the expression levels of NELFE and E2F2 in gastric cancer tissue were significantly positively correlated (**Figure 7C**).

### Correlation of NELFE and E2F2 With the Proportion of TICs

**Figure 8:**
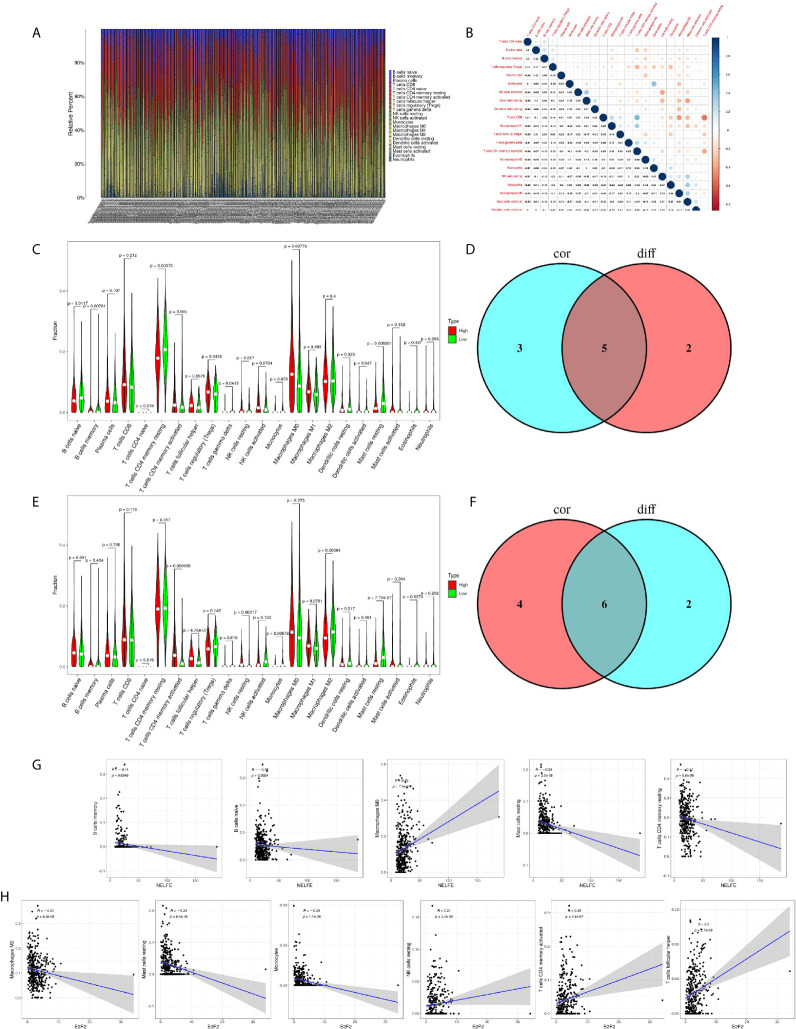
Correlation of NELFE and E2F2 with the proportion of TICs. TIC profile in tumor samples and correlation analysis. **(A)** Barplot showing the proportion of 22 kinds of TICs in gastric cancer samples. The column names in the plot are sample ID. **(B)** Heatmap showing the correlation between 22 kinds of TICs and numeric cells in each small box indicating the p value of the correlation between two kinds of cells. The shade of each small color box represents the corresponding correlation value between two cells, and the Pearson coefficient was used for the significance test. **(C)** Violin plot showing the ratio differentiation of 22 kinds of immune cells between gastric cancer samples with low or high NELFE expression relative to the median NELFE expression level, and Wilcoxon rank sum was used for the significance test. **(D)** Venn plot displaying five kinds of TICs correlated with NELFE expression codetermined by difference and correlation tests displayed in violin and scatter plots, respectively. **(E)** Violin plot showing the ratio differentiation of 22 kinds of immune cells between gastric cancer samples with low or high E2F2 expression relative to the median E2F2 expression level, and Wilcoxon rank sum was used for the significance test. **(F)** Venn plot displaying six kinds of TICs correlated with E2F2 expression codetermined by difference and correlation tests displayed in violin and scatter plots, respectively. **(G, H)** Scatter plot showing the correlation of the proportions of 5 kinds of TICs with NELFE expression and the proportions of 6 kinds of TICs with E2F2 expression.

To further confirm the correlation of NELFE and E2F2 expression with the immune microenvironment, the proportion of tumor-infiltrating immune subsets was analyzed using the CIBERSORTx algorithm. 22 kinds of immune cell profiles in STAD samples were constructed (**Figure 8A**). The correlation between different immune cells in gastric cancer tissue was objective (**Figure 8B**). The results from the difference and correlation analyses showed that a total of five kinds of TICs were correlated with the expression of NELFE (**Figures 8C, D**), and six kinds of TICs were correlated with the expression of E2F2 (**Figures 8E, F**). Among them, M0 macrophages were positively correlated with NELFE expression; four kinds of TICs were negatively correlated with NELFE expression, namely, memory B cells, naïve B cells, resting mast cells, and resting memory CD4+ T cells (**Figure 8G**). Three kinds of TICs were positively correlated with E2F2 expression, namely, resting NK cells, activated memory CD4+ T cells, and follicular helper T cells; Three kinds of TICs were negatively correlated with E2F2 expression, namely, M2 macrophages, resting mast cells and monocytes (**Figure 8H**). These results further supported the findings that the levels of NELFE and E2F2 affected the immune activity of the TME.

## Discussion

In our study, we systematically examined the promoting effect of an RNA-binding protein (NELFE) in human gastric cancer cells. Analysis of 224 cancer tissues and 224 adjacent nontumorous tissues indicated that NELFE was overexpressed in human gastric cancer compared with normal gastric tissues. Gastric cancer patients with high levels of NELFE showed both low OS rates and RFS rates. In BGC-823 and AGS gastric cancer cells, shRNA-mediated NELFE depletion dramatically decreased cell viability, as determined by the MTT assay and colony formation assay. *In vivo*, knockdown of NELFE inhibited the tumor formation ability in nude mice. In terms of metastasis, we also showed that knockdown of NELFE inhibited the metastasis ability *in vitro* and *in vivo*.

As reported previously, many RNA-binding proteins play important roles in the proliferation and metastasis of human cancer cells. Our team demonstrated that PCBP2 acted as an oncogene in gastric cancer and could promote the viability of human gastric cancer cells by regulating CDK2 (21). As a tandem zinc-finger RNA-binding protein, Loh et al. (22) proved that ZFP36L1 could markedly reduce bladder and breast cell proliferation *in vitro* and *in vivo* by suppressing hypoxia and cell cycle signaling. In terms of NELFE, Dang et al. (14) reported that NELFE promoted cell growth and metastasis of HCC. NELFE knockdown inhibited cell growth and metastasis both *in vitro* and *in vivo*. Furthermore, NELFE may cause HCC transcriptome imbalance by regulating MYC signaling and the NELFE-dependent MYC target (NDMT) gene signature to predict a unique subtype of HCC. These data all support the results obtained in our study. In addition, Han et al. (23) demonstrated that NELFE could promote proliferation, metastasis and EMT in pancreatic cancer. These results are in accordance with the data of the present study, which indicates that NELFE may have extensive carcinogenicity in tumors.

For the downstream pathway, E2F2 was chosen by RNA sequencing analysis. In this study, using an mRNA decay assay and a luciferase reporter assay, we found that the NELFE protein could bind to the E2F2 3’UTR and positively regulate E2F2 mRNA stability and protein expression. Exerting their biological activity by binding to RNAs is one of the functions of RNA-binding proteins. Zhao et al. (24) reported that RPS3 could promote HCC tumorigenesis both *in vitro* and *in vivo*. In addition, RPS3 stabilized SIRT1 mRNA by binding to AUUUA motifs on the 3’UTR of SIRT1 mRNA. In ovarian cancer, CELF2 could stabilize FAM198B mRNA by binding to AU/U-rich elements (AREs) in the 3’UTR (25). Moreover, ZFP36L1 bound to the HIF1A 3’UTR and mediated HIF1A mRNA degradation in bladder and breast cells (22).

Accordingly, E2F2 acts as an oncogene in gastric cancer. Abnormal overexpression or activation of E2F2 induces malignant cell viability. E2F2 was found to be overexpressed in human gastric cancer tissues compared with normal gastric tissues. In BGC-823 and AGS gastric cancer cells, E2F2 knockdown inhibited cell growth and metastasis both *in vitro* and *in vivo*. Rescue experiments showed that E2F2 overexpression abrogated the decrease in cell proliferation and metastasis by NELFE knockdown. In gastric cancer, E2F2 has not been systematically studied previously. E2F2 expression was reported to be upregulated in gastric cancer (26, 27), which is in accordance with the data of the present study. Additionally, H19 was demonstrated to promote gastric cancer cells to proliferate and invade through the miR-138/E2F2 axis (28). In cisplatin-resistant gastric cancer cells, miR-26a could improve the sensitivity of GC cells to cisplatin-based chemotherapies through E2F2 (29). All of these results support the findings of the present study. Moreover, E2F2 has been reported to promote tumor progression in many other human cancers, including breast cancer (30), ovarian cancer (31), lung cancer (32), and glioma (33). Therefore, through large number of *in vivo* and *in vitro* studies, we confirmed that NELFE facilitated the viability and metastasis of gastric cancer cells specifically by stabilizing the mRNA of E2F2. Herein, we propose a model to reveal the molecular mechanism of upregulation of E2F2 mRNA through NELFE in promoting gastric cancer pathogenesis, and it is illustrated in **Figure 7D**. The NELFE-E2F2 pathway was found to play an important role in human gastric cancer.

Recently, increasing evidence has shown that oncogenes and tumor suppressor genes could recruit or suppress different immune cells in the TME (34). TICs in the TME directly or indirectly promote tumorigenesis and the response to chemotherapy (18). It has been confirmed that the E2F family can recruit several immune cells in endometrial cancer (35) and CNS cancer (36). In the present study, we demonstrated that high expression of NELFE was positively correlated with M0 macrophages and that high expression of E2F2 was positively correlated with resting NK cells, activated memory CD4+ T cells, and follicular helper T cells. As a NELFE-E2F2 axis in gastric cancer, we were surprised to find that these two oncogenes played important roles in the infiltration of immune cells in gastric cancer. Combined with previous studies showing the relationship between TICs and tumor progression and drug resistance (18), we were able to conclude that the NELFE-E2F2 axis facilitated immune infiltration, which accelerated the proliferation and metastasis of gastric cancer. However, this finding needs to be verified in further research.

In summary, we have demonstrated that NELFE and E2F2 play a vital role in the proliferation and metastasis of gastric cancer, which may be mediated by effects on the tumor microenvironment. Enhanced NELFE and E2F2 expression levels were associated with poor survival rates in gastric cancer patients. NELFE and E2F2 inhibitors could be used as potential therapeutic targets for gastric cancer treatment. However, further study is needed to determine how the tumor microenvironment is regulated by NELFE and E2F2 or other RNA-binding proteins and to clarify the therapeutic value of these components.

## Data Availability Statement

All relevant data is contained within the article: The original contributions presented in the study are included in the article/**Supplementary Material**, further inquiries can be directed to the corresponding author/s.

## Ethics Statement

The studies involving human participants were reviewed and approved by Ethics Committee of the First Affiliated Hospital of Nanchang University. The patients/participants provided their written informed consent to participate in this study. The animal study was reviewed and approved by Animal Ethics Committee of Nanchang University. Written informed consent was obtained from the individual(s) for the publication of any potentially identifiable images or data included in this article.

## Author Contributions

CC, QZ and SP conducted the experiments, performed the data analysis and wrote the paper, WC, JH, YC, and YT performed experiments. CC and QZ analyzed data. ZL, CY and ZJ revised the manuscript, designed the experiment. All authors contributed to the article and approved the submitted version.

## Funding

This work was supported by the National Natural Science Foundation of China (No.81960503, 81860428), the Science and Technology Plan of Health Commission of Jiangxi Province (No.20191026), the Education Department of Jiangxi province science and technology research projects (No.GJJ160246), National Natural Science Foundation of Jiangxi Province (No.20202BABL216051), Key R&D general projects of the Science and Technology Department of Jiangxi Province (No.20203BBGL73187), Postgraduate Innovation Special Foundation of Jiangxi Province (YC2020-B054).

## Conflict of Interest

The authors declare that the research was conducted in the absence of any commercial or financial relationships that could be construed as a potential conflict of interest.
