# Supplementary figures and images for: The RNA-Binding Protein NELFE Promotes Gastric Cancer Growth and Metastasis Through E2F2
Source: Front Oncol. 2021 Jul 6;11:677111. doi: 10.3389/fonc.2021.677111 (PMC8290256; doi:10.3389/fonc.2021.677111)

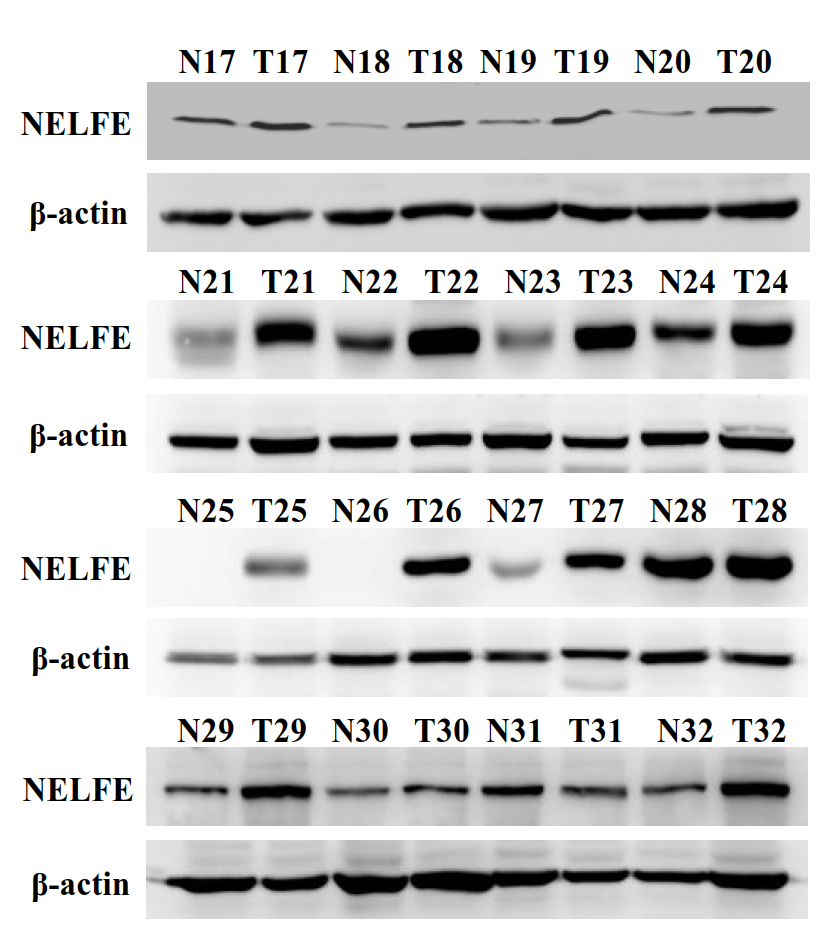

Supplement: Supplementary file 2 [file Image_1.tif]

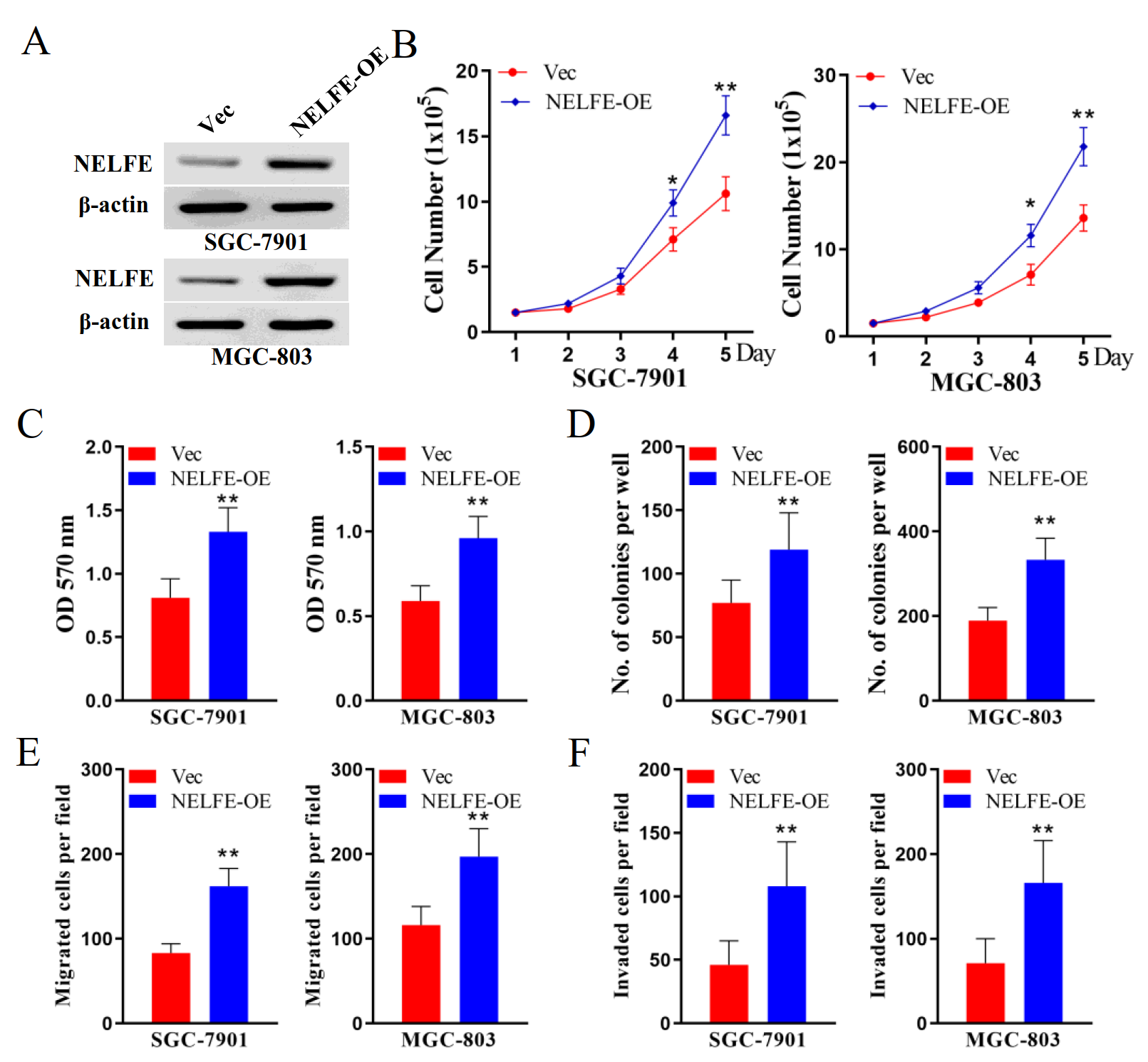

Supplement: Supplementary file 3 [file Image_2.tif]
