# Supplementary material for: The RNA-Binding Protein NELFE Promotes Gastric Cancer Growth and Metastasis Through E2F2
Source: Front Oncol. 2021 Jul 6;11:677111. doi: 10.3389/fonc.2021.677111 (PMC8290256; doi:10.3389/fonc.2021.677111)
Supplement: Supplementary file 4 [file Table_1.doc]

| Feature |  | Relative NELFE expression | | X^2^ | P Value |
| --- | --- | --- | --- | --- | --- |
|  |  | Low (109) | High (115) |  |  |
| Age | ≥ 60 | 51 | 63 | 1.1288 | 0.288 |
|  | < 60 | 58 | 52 |  |  |
| Gender | Male | 45 | 59 | 1.874 | 0.171 |
|  | Female | 64 | 56 |  |  |
| Tumor size (cm) | ≥ 5 | 47 | 75 | 10.146 | 0.001447 |
|  | < 5 | 62 | 40 |  |  |
| Differentiation | Poorly, undifferentiated | 43 | 67 | 7.1886 | 0.007337 |
|  | Well, moderately | 66 | 48 |  |  |
| Local invasion | T1+T2 | 54 | 48 | 1.077 | 0.2994 |
|  | T3+T4 | 55 | 67 |  |  |
| Lymph node  metastasis | Yes | 42 | 72 | 12.034 | 0.0005223 |
|  | No | 67 | 43 |  |  |
| TNM stage | I + II | 69 | 38 | 19.341 | 1.093e-05 |
|  | III + IV | 40 | 77 |  |  |

Table 1. Clinicopathologic correlation of NELFE expression in GC tissues
